# Association Between Chemotherapy-Induced Peripheral Neuropathy and Low Anterior Resection Syndrome

**DOI:** 10.3390/cancers16213578

**Published:** 2024-10-23

**Authors:** Samantha M. Linhares, Kurt S. Schultz, Nathan A. Coppersmith, Andrew C. Esposito, Ira L. Leeds, Haddon J. Pantel, Vikram B. Reddy, Anne K. Mongiu

**Affiliations:** Division of Colon and Rectal Surgery, Department of Surgery, Yale School of Medicine, New Haven, CT 06519, USAanne.mongiu@yale.edu (A.K.M.)

**Keywords:** low anterior resection syndrome, fecal incontinence, rectal neoplasm, chemotherapy-induced peripheral neuropathy

## Abstract

Introduction: Low anterior resection syndrome (LARS) can be a debilitating condition that develops after undergoing sphincter-preserving surgery for rectal cancer. Chemotherapy-induced peripheral neuropathy is a common side effect of platinum-based chemotherapy agents used as systemic therapy for rectal cancer treatment. The purpose of this study was to determine the potential relationship between CIPN and LARS. Methods: This was a retrospective review of patients who underwent a low anterior resection for rectal cancer and received systemic therapy contacted at least six months from the most recent surgery. Eligible patients were called and completed the relevant surveys over the phone or email. Results: There was a total of 42 patients who completed the surveys with 33 (79%) having major LARS. Presence of a diverting ileostomy was the only significantly differentcharacteristic in those with major LARS versus those without. CIPN was independently associated with LARS (*p* = 0.046) on linear regression when controlling for neoadjuvant chemoradiation, diverting ileostomy and tumor distance from the anal verge. Conclusions: Developing severe CIPN is associated with developing LARS. Further studies evaluating the etiology behind this relationship should be conducted.

## Simple Summary

Surgical treatment of rectal cancer with a low anterior resection allows patients to maintain normal anatomy. Despite this, many patients can postoperatively develop symptoms related to bowel dysfunction known as low anterior resection syndrome (LARS). Systemic therapy for rectal cancer treatment often includes platinum-based chemotherapy agents with peripheral neuropathy as a common side effect. LARS and chemotherapy-induced peripheral neuropathy (CIPN) may greatly affect a patient’s quality of life. Thus, the purpose of this study was to evaluate a potential relationship between CIPN and LARS. We found there was an association between CIPN and the development of LARS. Thus, further studies should look to evaluate the possible biological mechanisms behind this relationship.

## 1. Introduction

Over the past 20 years, advances in rectal cancer treatment have led to an increase in the rate of sphincter-preserving surgery, known as a low anterior resection (LAR). This has allowed many patients to avoid a permanent end colostomy [1,2,3]. However, despite restoring intestinal continuity, nearly 40–80% of patients who undergo an LAR will develop lower anterior resection syndrome (LARS) [4,5]. LARS refers to a constellation of symptoms, comprising one of eight symptoms after sphincter sparing surgery including (1) variable, unpredictable bowel movements, (2) emptying difficulties, (3) altered stool consistency, (4) urgency, (5) increased stool frequency, (6) incontinence, (7) repeated painful stools, and (8) soiling, resulting in at least of one of the eight following consequences: (1) toilet dependence, (2) preoccupation with bowel function, (3) dissatisfaction with bowels, (4) strategies and compromises, (5) impact on mental and emotional wellbeing, (6) impact on social and daily activities, (7) impact on relationship and intimacy, and (8) impact on roles, commitments, and responsibilities [6,7]. Despite the high prevalence of LARS, it is not represented by any ICD-10 code and often goes overlooked and undertreated [8,9].

The etiology behind LARS is multifactorial with contributing factors including intestinal hypermobility due to both a neo-rectum and damage to the normal pelvic innervation during total mesorectal excision [4,10,11,12]. Additional risk factors have been identified including neoadjuvant chemoradiation, low anastomotic height, requiring a diverting ileostomy at the index operation, and anastomotic leak [4,13,14]. Prior efforts to alleviate LARS symptoms using different surgical techniques such as a colonic reservoir did not prove to provide superior results compared to the typical side-to-side anastomosis [15,16]. Many of these risk factors are immutable, making LARS an unavoidable postoperative consequence.

Patients with locally advanced rectal cancer often receive adjuvant or neoadjuvant platinum-based chemotherapy. Chemotherapy-induced peripheral neuropathy (CIPN) is defined as sensory, motor, or autonomic symptoms that are a common side effect of systemic treatment affecting nearly 60–87% of patients, with 30% experiencing ongoing neuropathy six months or more after completion of treatment [17]. There are few effective evidence-based treatments for CIPN [18,19]. Consequently, both CIPN and LARS can become chronic conditions with detrimental effects on patients’ QoL following rectal cancer treatment. Given that nerve damage is suspected to contribute to LARS, patients who are more vulnerable to developing CIPN might suffer from a compounded effect. This could be contributing factor to LARS that has not yet previously been explored. Thus, we aimed to evaluate whether there was an association between CIPN and major LARS in patients who underwent systemic therapy and LAR.

## 2. Materials and Methods

This was a retrospective study of patients with rectal cancer who received chemotherapy and an LAR between January 2017 and December 2023 at a single, university-affiliated health system. There were four board-certified colorectal surgeons who performed the surgeries. Patients were identified using the ICD-10 code C20 (“malignant neoplasm of the rectum”). Patients were contacted at least six months postoperatively from the restoration of gastrointestinal continuity (from ostomy closure or LAR if there was no index diversion). All patients received a platinum-based chemotherapy regimen either in the neoadjuvant or adjuvant setting. Non-English-speaking patients were excluded. Patients were contacted and completed a structured interview by phone or email.

Questionnaires included the LARS score, which is a validated self-administered questionnaire composed of five questions that address the five key symptoms of LARS: incontinence of stool, incontinence of flatus, frequency, clustering, or urgency related to bowel movements. The severity of LARS is scored along a scale of 0–42 with no LARS equating to a score of 0–20, minor LARS as a score between 21 and 29 and major LARS as a score from 30 to 42 [6]. The European Organization for Research and Treatment of Cancer Quality of Life Questionnaire Chemotherapy-Induced Peripheral Neuropathy-20 (EORTC QLQ-CIPN20) is also a validated scale composed of twenty questions that measure three different scales of neuropathy including sensory (numbness, tingling, burning or pain in fingers, or toes), motor (difficulty holding or picking up objects), and autonomic (orthostatic hypotension, getting and/or maintaining an erection) symptoms and function with a score ranging from 0 to 100 (Supplementary Figure S1) [20]. Clinical information including age, sex, tumor characteristics, and final pathology was extracted from the electronic medical record.

Distributions of categorical variables were compared using Chi-squared tests and continuous variables using *t*-test or Wilcoxon rank-sum tests as appropriate. Linear regression and Spearman’s rank correlation coefficients were used to evaluate the association of CIPN and LARS scores. Statistical analyses were performed using SPSS version 24.0 (International Business Machines, Chicago, IL, USA). The level of significance was set at *p* < 0.05. Findings were reported per guidelines established by the Strengthening the Reporting of Observational Studies in Epidemiology (STROBE) Statement. The Institutional Review Board deemed the study exempt. Informed consent was waived as this was a retrospective study posing minimal risk to included subjects.

## 3. Results

Sixty-five eligible patients were contacted with 42 patients completing the questionnaires (65% response rate). There were 33 (79%) patients suffering from major LARS. The patient population had a mean age of 59.4 (SD 10.9) with 17 (41%) female patients and an average BMI of 26.8 (SD 4.9). The final pathologic stage after resection ranged from complete pathologic response to stage 3. Demographic characteristics were not statistically significant among patients with major LARS and those without. Similarly, there was no significant difference in rates of neoadjuvant chemoradiation (88% versus 67%, *p* = 0.155), adjuvant chemotherapy (21% versus 0%, *p* = 0.123), and mean tumor distance from the anal verge (9.4 cm versus 9 cm, *p* = 0.809). Compared to those without major LARS, patients with major LARS had higher incidences of diverting loop ileostomy (97% versus 67%, *p* = 0.021). However, the median length of duration of diverting ileostomy was significantly greater for patients with no or minor LARS (7 months versus 5 months, *p* = 0.032 Table 1). There were no incidents of anastomotic leak in the overall cohort.

**Figure 1:**
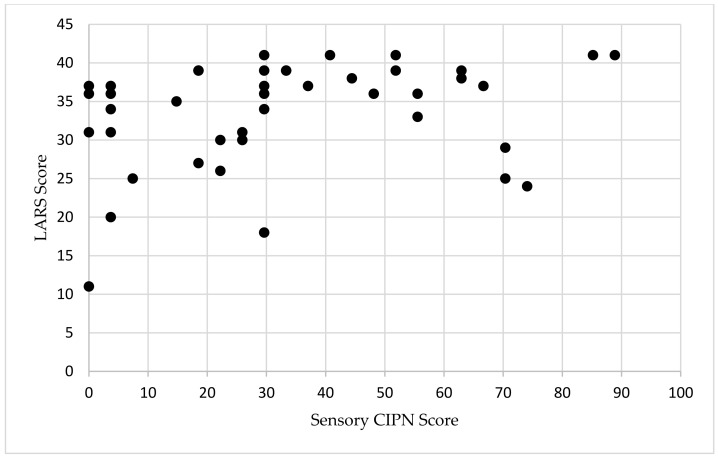
Scatterplot of LARS and sensory CIPN in patients with rectal cancer. LARS scores range from 0 to 42. Sensory CIPN scores range from 0 to 100. Abbreviations: LARS, low anterior resection syndrome; CIPN, chemotherapy-induced peripheral neuropathy.

For patients with major LARS, the median CIPN score was 29.6 (IQR 17–52) and was 22 (IQR 5.6–70) in patients without major LARS (Table 1). The median time since most recent surgery (either diverting loop ileostomy reversal or index LAR without diversion), was 37 months for patients with major LARS and 36 months for patients without major LARS (*p* = 0.526). The Spearman’s rank order correlation showed a weak correlation between sensory CIPN and the LARS score (r(s) = 0.322, *p* = 0.033, Figure 1). On multivariable linear regression controlling for neoadjuvant chemoradiation, undergoing a diverting stoma, and distance from anal verge, CIPN was independently associated with LARS (*t*-statistic 2.07, *p* = 0.046, Table 2). There was no correlation between LARS score and the time of survey completion (*p* = 0.941, Supplementary Figure S1).

## 4. Discussion

Prior studies and our results show that LARS is experienced by a large proportion of patients following treatment for rectal cancer. Pelvic nerve damage is thought to arise from total mesorectal excision and results in increasing colonic motility [11]. No prior studies have investigated a potential relationship between the severity of CIPN and LARS score. We hypothesized that severe CIPN may contribute to LARS. Our results show that CIPN is a significant predictor of LARS in patients with rectal cancer who underwent systemic therapy and sphincter-sparing surgery.

Multiple risk factors for developing LARS have been identified including neoadjuvant chemoradiation, low anastomosis, requiring a diverting stoma, or anastomotic leak [4,10]. Both Hughes et al. and Stuirale et al. found that an anastomosis less than five cm from the anal verge was significantly associated with major LARS [14,21]. In our study, there was no significant difference between rates of neoadjuvant chemoradiation or tumor distance from anal verge between those with major LARS and those without. While 88% of patients with major LARS received neoadjuvant chemoradiation compared to 67% of patients without major LARS, the small sample size may have contributed to the lack of statistical significance. In our patient population, there was a significantly higher rate of diverting stoma for those with major LARS, although those without major LARS had a statistically significant longer duration of diverting ileostomy. This finding contradicts multiple other studies, but given our small sample size, this finding should be interpreted with caution. Disuse atrophy of the pelvic floor musculature from a longer duration of diverting ileostomy has been suggested to increase the risk of LARS [14,21]. The recommended timing for diverting loop ileostomy reversal is before 90 days to reduce risk of complications [22]. Adoption of total neoadjuvant therapy (TNT) has been shown to increase the feasibility of earlier ileostomy reversal [23]. Given that TNT was only widely adopted at our institution around 2021, this may have contributed to the longer stoma duration and high incidence of major LARS in our patient population [24]. There has been evidence to suggest that stoma reversal as soon as 8–13 days after an operation without signs of anastomotic leak is safe, and could potentially be adopted as a method to reduce incidence of LARS [25]. Lastly, sex as a risk factor for LARS has mixed results within the literature, and it was not a significant factor in our study [4,13,14,26].

Prior studies have shown that improvement in LARS typically occurs within the first year after surgery; however, between 45 and 58% of patients still suffer from major LARS after one year [27,28]. Patients may experience some further improvement after one year but they are significantly less likely to undergo substantial change [21]. In our study population, 80% of patients still had major LARS 12 months after their last surgery [5,29]. Additional studies in 2015 and 2017 showed that approximately 50% of patients more than 10 years after their operation still suffered from major LARS [21,29]. This suggests that there is an opportunity to help improve patients’ QoL by mitigating symptoms using an escalating treatment algorithm, starting with conservative treatments including anti-diarrheal medications and dietary changes, followed by a consideration of pelvic floor rehabilitation and trans-anal irrigation, or a consideration of sacral nerve stimulation or conversion to end colostomy [30].

The limitations of our study include the design as a single-center retrospective study with a small study size. Given that 35% of patients did not respond, non-response bias may have led to an overrepresentation of patients with major LARS. Due to the remote timing of the study compared to when patients were undergoing treatment, this narrowed the possible sample size and may have contributed to the non-response bias. In addition, this study is lacking in longitudinal data to track symptomology. Next steps would be to compare bowel function preoperatively and postoperatively to best evaluate how bowel function changes over time, as prior studies have shown that patients can score as having “major LARS” on survey completion without undergoing an LAR [31]. CIPN has been shown to have the highest incidence after the first cycle of chemotherapy and so capturing this data prospectively would also be beneficial [17].

Based on the results from this study, there may be a common nerve injury pathway contributing to both CIPN and LARS. Currently, there are limited recommendations for the treatment of CIPN as per multiple societal guidelines with no identified treatments for numbness/tingling. The only treatment known to be moderately effective for neuropathic pain is duloxetine, a serotonin and norepinephrine reuptake inhibitor [17]. Serotonin receptor antagonists have also been investigated in LARS and have been shown to have some efficacy for symptom improvement [32]. Further research could evaluate the underlying mechanism behind this. This study also highlights the long-term persistence of LARS, and attempting to mitigate the development of this syndrome continues to be a challenge. There are no validated risk calculators used to preoperatively identify high-risk patients for developing LARS. One nomogram has been developed called the Pre-Operative LARS score (POLARS), which scores bowel dysfunction severity; however, a follow-up retrospective study attempting to measure this score found that this score had low sensitivity [33,34]. Further efforts to develop an effective predictive tool should be investigated. A growing interest involves engaging patients prior to surgery with prophylactic therapy, known as prehabilitation, and has been shown to reduce postoperative complications and improve functional outcomes [35,36]. The use of pelvic floor rehabilitation, shown to be effective in the postoperative period, could be developed for prehabilitation to reduce the incidence of LARS [37,38,39]. Solutions for these gaps in treatment should be further investigated in future studies.

## 5. Conclusions

In this study, there was an association between higher CIPN and major LARS in patients undergoing multimodal treatment for rectal cancer. Both of these conditions can greatly affect a patient’s quality of life. There was a high incidence of major LARS persisting beyond one year after surgery, highlighting the need for early and timely intervention for treatment. Investigating of the biological mechanisms underlying sensory CIPN and LARS could identify interventions to mitigate their effects on rectal cancer recovery.

## Figures and Tables

**Table 1:** Demographic characteristics of study population.

Characteristics	Overall Cohort (*n* = 42)	Major LARS (*n* = 33)	No/Minor LARS (*n* = 9)	*p*-Value
Age, years, mean (SD)	59.4 (10.9)	59.6 (10.6)	60 (26.8)	0.914
Sex, female, *n* (%)	17 (41)	12 (37)	5 (56)	0.446
BMI, kg/m^2^, mean, (SD)	26.8 (4.9)	26.8 (4.6)	26.8 (6.4)	0.976
Pathologic stage, *n* (%)				0.438
Complete pathologic response/in situ, *n* (%)	12 (29)	10 (30)	2 (22)	
Stage 1	10 (24)	9 (27)	1 (11)	
Stage 2	6 (14)	5 (16)	1 (11)	
Stage 3	14 (33)	9 (27)	5 (56)	
Neoadjuvant chemoradiation, *n* (%)	35 (83)	29 (88)	6 (67)	0.155
Neoadjuvant chemotherapy, *n* (%)	7 (17)	7 (21)	0 (0)	0.123
Diverting ileostomy at time of LAR, *n* (%)	38 (91)	31 (97)	6 (67)	0.006
Duration of diverting ileostomy, months, median (IQR)	5.5 (3.5–7)	5 (2–6)	7 (5.8–8.5)	0.032
Distance from anal verge, cm, mean (SD)	9.3 (4.6)	9.4 (4.4)	9 (5.5)	0.809
Time since most recent surgery, months, median (IQR)	36.5 (15–50)	37 (16.5–51)	36 (12.5–50)	0.526
Sensory CIPN score, median (IQR)	29.6 (13–53)	29.6 (17–52)	22 (5.6–70)	0.833

Abbreviations: LARS, low anterior resection syndrome; SD, standard deviation; BMI, body mass index; IQR, interquartile range; CIPN, chemotherapy-induced peripheral neuropathy.

**Table 2:** Multivariable linear regression of LARS score.

Variable	Standardized Coefficients (Beta)	Standard Error	T-Statistic	*p*-Value
Sensory CIPN score	0.27	0.036	2.07	0.046
Diverting loop ileostomy	0.57	4.5	2.9	0.006
Neoadjuvant chemoradiation	0.02	3.5	0.08	0.94
Distance from anal verge	0.16	0.21	1.2	0.26

Abbreviations: CIPN, chemotherapy-induced peripheral neuropathy.

## Data Availability

The original contributions presented in this study are included in the article and further inquiries can be directed to the corresponding author.

## Supplementary Materials

The following supporting information can be downloaded at: https://www.mdpi.com/article/10.3390/cancers16213578/s1; Supplementary Figure S1. Scatterplot of length of time from last surgery and LARS score.
