# Supplementary material for: Association Between Chemotherapy-Induced Peripheral Neuropathy and Low Anterior Resection Syndrome
Source: Cancers (Basel). 2024 Oct 23;16(21):3578. doi: 10.3390/cancers16213578 (PMC11545659; doi:10.3390/cancers16213578)
Supplement: Supplementary file 1 [file cancers-16-03578-s001.zip › cancers-3208174-supplementary.pdf]

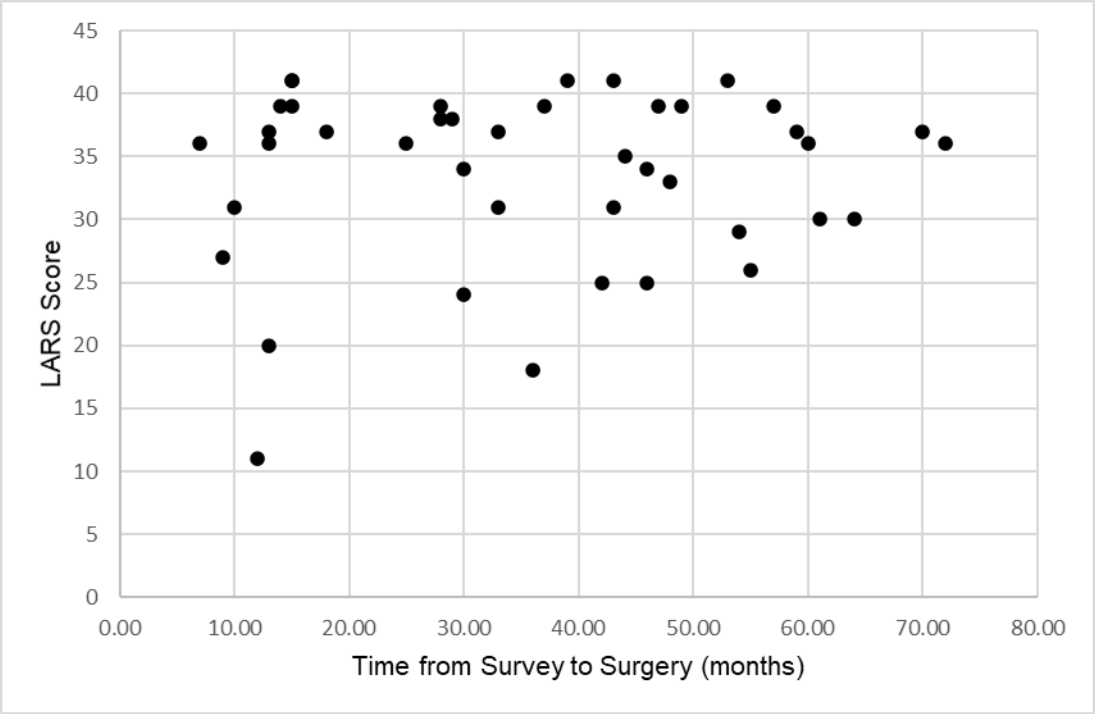

Supplementary Figure S1. Length of time from last surgery compared to LARS score. Abbreviations: LARS = low anterior resection score.
